# Risk Factors of Dengue Fever in Urban Areas of Rawalpindi District in Pakistan During 2017: A Case Control Study

**DOI:** 10.2196/27270

**Published:** 2022-01-19

**Authors:** Najma Javed Awan, Ambreen Chaudhry, Zakir Hussain, Zeeshan Iqbal Baig, Mirza Amir Baig, Rana Jawad Asghar, Yousef Khader, Aamer Ikram

**Affiliations:** 1 Field Epidemiology and Laboratory Training Program National Institute of Health Pakistan Islamabad Pakistan; 2 Global Health Strategists and Implementers Islamabad Pakistan; 3 Department of Community Medicine, Public Health and Family Medicine Faculty of Medicine Jordan University of Science & Technology Irbid Jordan; 4 National Institute of Health Pakistan Islamabad Pakistan

**Keywords:** dengue fever, outbreak, Rawalpindi, risk factors, stored water, urban

## Abstract

**Background:**

During August 2017, increased numbers of suspected dengue fever cases were reported in the hospitals of Rawalpindi district. A case control study was conducted to determine the risk factors among urban areas, dengue serotype, and recommend preventive measures.

**Objective:**

The objective of the investigation was to determine the risk factors among urban areas, dengue serotype, and recommend preventive measures.

**Methods:**

A case was defined as having acute febrile illness with one or more of the following symptoms: retro-orbital pain, headache, rash, myalgia, arthralgia, and hemorrhage. The cases were residents of Rawalpindi and were confirmed for dengue fever from August 30, 2017, to October 30, 2017. All NS1 confirmed cases from urban areas of Rawalpindi were recruited from tertiary care hospitals. Age- and sex-matched controls were selected from the same community with a 1:1 ratio. Frequency, univariate, and multivariate analyses were performed at 95% CI with *P*<.05 considered statistically significant.

**Results:**

Totally 373 cases were recruited. The mean age was 36 (SD 2.9) years (range 10-69 years), and 280 cases (75%) were male. The most affected age group was 21-30 years (n=151, attack rate [AR] 40%), followed by 31-40 years (n=66, AR 23%). Further, 2 deaths were reported (case fatality rate of 0.53%). The most frequent signs or symptoms were fever (n=373, 100%), myalgia and headache (n=320, 86%), and retro-orbital pain (n=272, 73%). Serotype identification was carried out in 322 cases, and DEN-2 was the dominant serotype (n=126, 34%). Contact with a confirmed dengue case (odds ratio [OR] 4.27; 95% CI 3.14-5.81; *P*<.001), stored water in open containers at home (OR 2.04; 95% CI 1.53-2.73; *P*<.001), and travel to a dengue outbreak area (OR 2.88; 95% CI 2.12-3.92; *P*<.001) were the main reasons for the outbreak, whereas use of mosquito repellents (OR 0.12; 95% CI 0.09-0.18; *P*<.001) and regular water supply at home (OR 0.03; 95% CI 0.02-0.04; *P*<.001) showed protective effects. The geographical distribution of cases was limited to densely populated areas and all the 5 randomly collected water samples tested positive for dengue larvae.

**Conclusions:**

Stored water in containers inside houses and subsequent mosquito breeding were the most probable causes of this outbreak. Based on the study findings, undertaking activities to improve the use of mosquito repellents and removing sources of breeding (uncovered water stored indoors) are some recommendations for preventing dengue outbreaks.

## Introduction

Dengue is a viral infection that is transmitted to the host by the mosquito vector Aedes aegypti. Symptoms vary from flu-like ones to potential lethal complications including hemorrhages. Currently, there are 4 distinct serotypes of the virus that are identified as causing dengue (DEN-1, DEN-2, DEN-3, and DEN-4). Infection from one serotype provides lifelong immunity against that serotype [1]. Clinical manifestations of dengue virus infection range from asymptomatic infection to dengue fever (DF), dengue hemorrhagic fever, or dengue shock syndrome, and these may affect other organs such as the liver, kidneys, brain, or heart [2,3].

Approximately 390 million dengue infections occur annually. However, only 96 million infections manifest clinically [4]. An estimated 3.9 billion people are at risk of contracting this disease worldwide [5]. Since 1994, Pakistan is facing dengue outbreaks [6,7]. The first confirmed case of DF in Pakistan was reported in Karachi city in 1994 [8]. There has been a dramatic rise in dengue cases, and numbers have increased from 4500 cases in Karachi in 2005 to 21,204 cases in 2010 nationally. During 2011, there were 14,000 confirmed cases and 300 deaths in Lahore district only due to DF. However, even these data do not portray the true situation in the country, as the actual burden is expected to be much higher than reported [9]. Later, in 2018, DF was added to the list of priority diseases in Pakistan [10]. In 2019, 19,000 cases were reported at the National Institute of Health [11] and the toll rose to 52,000 until a Public Health Emergency Operations Center coordinated with all departments to control the outbreak 2 weeks earlier compared to the previous year’s outbreaks [12]. In 2020, the case burden in Pakistan tripled, including COVID-19, measles, and DF [13]. As no specific medicine or vaccine has been developed for DF, the only method to control this disease is through prevention (vector control) using long-lasting insecticide-treated materials effective for more than 5 years. Similarly, homes, offices, and schools can be protected from Aedes aegypti using bed and window nets, which is the cheapest method of controlling the disease [14,15]. Hospital admissions for dengue infection start increasing from August (monsoon season in Pakistan), and the same pattern is prevailing in other neighboring countries such as India and Bangladesh [16].

During August 2017, an outbreak was announced by the health authorities of Rawalpindi district. To design and employ effective preventive and control strategies against the disease, it was necessary to identify the risk factors of the disease prevailing in the district and share these results with the public health authorities for targeted control strategies.

**Figure 1:**
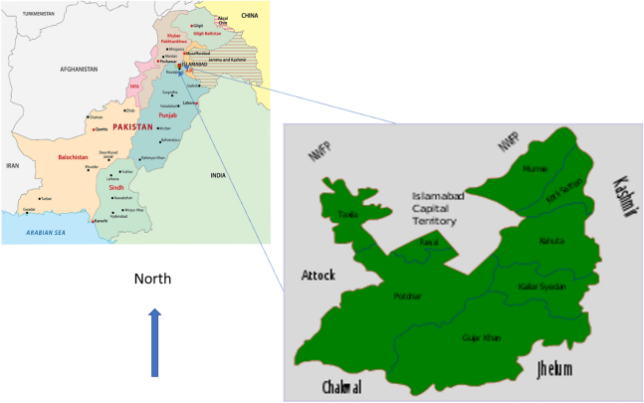
Geography of district Rawalpindi, Pakistan.

This investigation was conducted to determine the risk factors associated with DF among patients from urban areas of Rawalpindi to estimate the prevalent serotype in this outbreak and recommend measures for prevention. Rawalpindi is a metropolitan city neighboring the capital Islamabad, and as the disease is considered an urban disease, we decided to examine these factors among dengue cases coming from the urban areas of this district. Figure 1 shows the geographical location of Rawalpindi district.

## Methods

Records of the tertiary care hospitals of the district were obtained, and the history of recent influxes of migrants like internally displaced population was also ruled out.

A case control study was designed to determine the risk factors associated with this disease. All patients visiting the tertiary care hospitals of Rawalpindi with acute febrile illness and any 3 symptoms among retro-orbital pain, headache, rash, myalgia, arthralgia, and hemorrhagic manifestations between August 30 and October 30, 2017, were admitted according to the guidelines provided by the provincial health department [17]. Blood samples were collected from the patients enrolled according to the criteria set by the Public Health Laboratory Division of the National Institute of Health and were tested for dengue IgM, IgG, and NS1.

All laboratory-confirmed cases were recruited from the inpatient departments of the hospitals. A functional case was defined as the onset of acute febrile illness with one or more of the following symptoms: retro-orbital pain, headache, rash, myalgia, arthralgia, and hemorrhagic manifestations from August 30 to October 30, 2017, which was in accordance with the case definition established by the Department of Health. The residential addresses of the patients were collected, and age- and sex-matched controls were enrolled from the same community with a 1:1 ratio. The controls were defined as residents from the neighborhood of the cases who had not experienced acute febrile illness from August 30 to October 30, 2017, and had not been diagnosed as having DF by any physician or laboratory during this time.

An institutional review board exception was obtained from the National Institute of Health in Islamabad. After obtaining informed written consent translated to Urdu and reading out the same to the respondents where necessary, a close-ended, structured, and pretested questionnaire was used to collect data from cases and controls regarding general characteristics and possible risk factors (Multimedia Appendix 1). Information was collected on indoor or outdoor insecticidal sprays within the last 10 days in their area. Water samples were collected from 5 randomly selected places with stagnant water and from water stored indoors for detection of larvae. Water samples were sent to the Institute of Public Health in Lahore for dengue larvae detection. Samples were also collected for serotyping and sent to the provincial laboratory of the Institute of Public Health with permission from the district health authorities.

Frequency, univariate, and multivariate analyses were performed using statistical software Epi Info 7 (Centers for Disease Control and Prevention). An epidemic curve was constructed to demonstrate the distribution of cases over time. The cases were plotted on a spot map to understand the geographical distribution of the cases in the area. Age- and gender-wise infection rates were calculated. The odds ratios (ORs) were calculated for different exposures at 95% CI and *P*<.05 was considered statistically significant.

## Results

**Figure 2:**
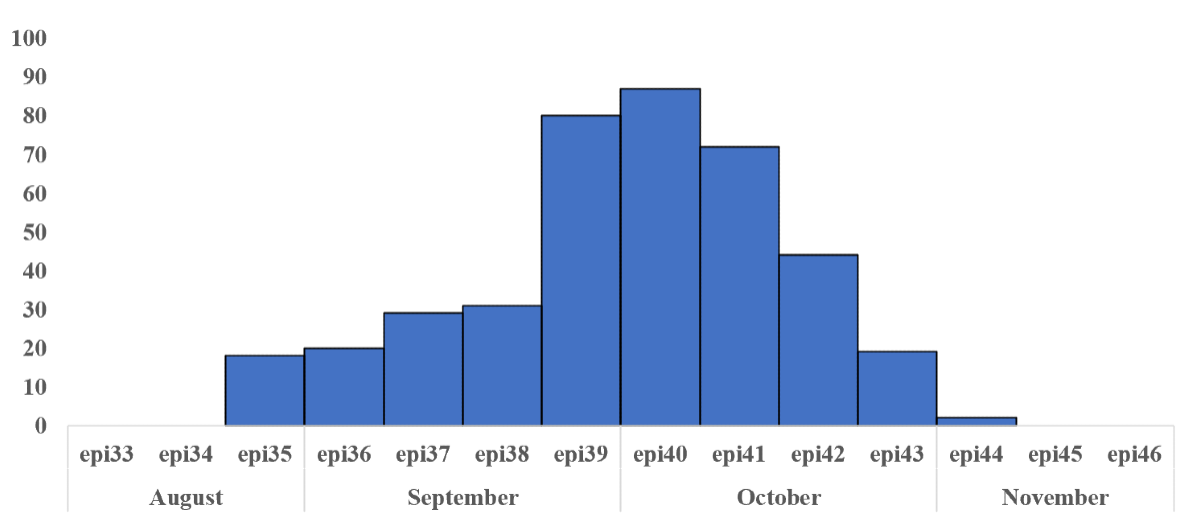
Epidemic curve showing the time distribution of dengue cases in Rawalpindi during 2017 (N=373).

The outbreak started on August 29, 2017, and it started declining on October 30, 2017, peaking during the last week of September and first week of October, as shown in Figure 2. Totally 373 cases were enrolled from tertiary care hospitals, as confirmed by their respective laboratories through NS1 tests.

The mean age of the confirmed cases was 36 (SD 2.9) years (range 10-69 years) with a male-to-female ratio of 3:1. Most of the cases were in the age group of 21 to 30 years (n=151, attack rate [AR] 40%), followed by the age group of 31 to 40 years (n=66, AR 23%). Further, 2 deaths were reported (case fatality rate=0.53%). The most frequent symptom was fever (n=373, 100%), followed by myalgia (n=320, 86%), headache (n=320, 86%), and retro-orbital pain (n=272, 73%). Serotype identification was carried out for 322 cases. DENv-2 (n=126, 39%) was the most prevalent serotype followed by DENv-3 (n=96, 30%), DENv-4 (n=58, 18%), and DENv-1 (n=42, 13%). Table 1 presents the statistics.

Most patients had leukopenia (mean 4.5 [SD 5.06]) whereas hemoglobin levels were within normal limits (13.76 [SD 2.5]).

Out of the 373 confirmed cases, 237 were contacts of a confirmed case (OR 4.27; 95% CI 3.14-5.81; *P*<.001) and 219 stored water in open containers (OR 2.04; 95% CI 1.53-2.73; *P*<.001). Further, 189 people traveled to an area with dengue outbreak (OR 2.88; 95% CI 2.12-3.92; *P*<.001). Regular water supply at home (OR 0.03; 95% CI 0.02-0.04; *P*<.001) and regular use of mosquito repellents (OR 0.12; 95% CI 0.09-0.18; *P*<.001) proved effective in preventing dengue. In contrast, previous visits to hospitals (OR 0.83; 95% CI 0.57-1.21; *P*=.34) showed no significant association with dengue infection, as observed in Table 2.

The geographical distribution of dengue cases showed the typical characteristics of dengue mosquitos, limiting their activity within pockets of densely populated areas and avoiding crossing of highways in urban dwellings. Water samples taken from 5 randomly selected stagnant water places and from water stored indoors for detecting larvae tested positive for larvae.

**Table 1:** Statistics of dengue cases in Rawalpindi during 2017 (N=373).

Characteristics		n (%)
**Sex**		
	Male	278 (75)
	Female	95 (25)
**Age group (years)**		
	10-20	96 (26)
	21-30	151 (40)
	31-40	66 (18)
	41-50	39 (10)
	≥50	21 (7)
**Signs or symptoms**		
	Fever	373 (100)
	Myalgia	320 (86)
	Headache	320 (86)
	Retro-orbital pain	272 (73)
**Serotype (n=322)**		
	DENv^a^-1	42 (13)
	DENv-2	126 (39)
	DENv-3	96 (30)
	DENv-4	58 (18)

^a^DENv: dengue virus

**Table 2:** Factors associated with dengue infection among residents of Rawalpindi during 2017 (N=373).

Risk factors	Cases	Controls	OR^a^	95% CI	*P* value
Contact with a confirmed case	237	108	2.35	3.14-5.81	<.001
Stored water in open containers at home	219	153	2.04	1.53-2.73	<.001
Travel to areas with dengue outbreak	189	98	2.88	2.12-3.91	<.001
Regular water supply at home	64	322	0.03	0.02-0.04	<.001
Regular use of mosquito repellant	48	199	0.12	0.09-0.18	<.001
Previous visit to a hospital	300	310	0.83	0.57-1.21	.34

^a^OR: odds ratio.

## Discussion

### Principal Findings

This study showed that males were more affected than females, and the young age group of 21 to 30 years was the most severely affected (AR=40%). Stored water in containers inside houses and subsequent mosquito breeding were the most probable causes of the outbreak and the use of mosquito repellents had a protective effect. Dengue affects all age groups including infants and adults [17]; however, children usually tolerate this infection better than adults [18]. Our results support this finding, as there were only 3 children under 10 years of age admitted during this outbreak and none in infancy.

Simmons et al found that in mild dengue cases, laboratory analysis shows no significant changes except for abnormal leukocyte counts and moderate elevation of the hepatic amino-transferase enzyme activity [19]. This phenomenon was observed in our study too, where there was no significant difference between the laboratory parameters of the cases and controls.

In our study, the case fatality rate was 0.53%, showing that timely medical care and symptomatic management saved lives. Gubler states that the case fatality can be reduced to less than 1% with correct and timely treatment [20]. Akhter emphasizes that even patients with complications can be cured if given supportive and adequate treatment [21]. This explains the low case fatality during this outbreak, as the government had referral hospitals (Holy Family Hospital, Rawalpindi) and had devised the diagnosis and management criteria for all suspected, confirmed, and complicated DF cases at primary and secondary care hospitals.

According to the classification schemes of the World Health Organization, leukopenia in patients with febrile illness is one of the key findings when suspecting dengue infection [22]. In the present study, most of the patients presented low leukocyte levels and relatively better hemoglobin levels. The average leukocyte count was 4.5 among the admitted dengue patients. Other studies have documented that case fatality rates of dengue increase when infection occurs in patients with other acute or chronic diseases like asthma, diabetes, and hypertension [23,24].

Vector control is crucial in preventing DF. Along with the availability of impregnated bed nets, other measures like window curtains and water container covers treated with long-lasting insecticide have been tested in dengue endemic countries [25]. Only 48 individuals out of 373 were using mosquito repellents or any kind of protection against mosquitos; however, in the control group, 199 used mosquito repellents and this proved protective.

There are 4 distinct dengue virus serotypes that cause dengue (DEN-1, DEN-2, DEN-3, and DEN-4) [1]. During this outbreak, 322 blood samples were tested. DEN-2 (n=126, 39%) was the most prevalent serotype, followed by DEN-3 (n=96, 30%), DEN-4 (n=58, 18%), and DEN-1 (n=42, 13%). In previous outbreaks of dengue reported from different cities of Pakistan, DEN-2 remained the prominent serotype. In the dengue outbreaks in 2008 and 2009, DEN-2, 3, and 4, and DEN-2 and 3 were prominent, respectively [26]. Similarly, according to dengue case data from Sheikhupura and Gujranwala districts, DEN-2 was the most prevalent, followed by the DEN-1 serotype 1 [27].

Most of the cases were males with a male-to-female ratio of 3:1. This finding confirms those of previous studies [28,29]. Male predominance may be due to multiple reasons. Males are usually responsible for taking children early in the morning to school, and they go out for work. They are also responsible for bringing food and other items in the evening. As it was summer, males usually wore thin clothes with half sleeves, thus becoming more vulnerable to mosquito bites. In comparison, females stay at home and according to the local culture, they are well covered. Fatima reported the same findings where 73% of the cases comprised males and the mean age of the subjects was 34 years with a range of 5 to 80 years [30]. Similar results were obtained in our investigation where the mean age was 36 years (range 10-69 years).

The presence of stored water in homes, usually in open containers, for domestic use was observed because of intermittent water supply. Storing water was found to be a risk factor for spreading DF. Out of the 373 dengue cases, 307 had intermittent water supply and 219 were storing water at home for domestic and drinking purposes (OR 2.04, *P*<.001), and 196 had stagnant water pools, ponds, or passages near their homes. Fatima reported that the source of water supply is a risk factor for DF [30]. This finding supports the findings of another study from Vietnam [31]. This study also states that the absence of taps was strongly associated with DF. Apart from stored water in homes, open wells were the major source of water supply for the study population and both factors promoted vector breeding. Phoung et al highlight the same issue, identifying that mosquito larvae in water containers and gardens near houses are the most important risk factors for dengue transmission [32].

Recently, Wang et al have described that there are several risk factors that correlate with dengue hemorrhagic fever, including viral, epidemiological, human, and abiotic factors [33]. Another study conducted among young children has revealed the same risk factors as those identified in our study. Among the people in the study population, those storing water in their homes and consistently covered the storage containers did not develop dengue as opposed to those who did not. Similarly, the positivity of the dengue virus was significant (*P*<.001) among children who did not regularly wear long-sleeved shirts and full pants [34].

Consequently, different prevention and control activities were performed during the outbreak, including awareness campaigns about DF, filling of stagnant water reservoirs, and discouraging water storage at home. Insecticide-treated bed nets were distributed, and their use was demonstrated.

### Recommendations

1. Enhance community health sessions to increase awareness about DF and its preventive measures among the general public.
2. District administrations must prioritize filling of stagnant water reservoirs and discourage water storage in open containers.
3. Promote the use of mosquito repellents.
4. Provide and distribute impregnated bed nets and demonstrate their use.
5. Sensitize the local community elders, schoolteachers, and influential persons about the seriousness of the issue and obtain their support.

### Limitations

Owing to time constraints and limited monetary resources, all environmental and serotyping tests were not carried out.

### Conclusions

Dengue is a re-emerging disease. There are multiple factors that can contribute to the development of this disease. Owing to the overall change in the global environment and deteriorating conditions like poverty, access to basic necessities of life, health care, and conflicts in most of the developing countries, dengue is now an endemic disease. More focused studies will be required to pinpoint the risk factors along with efforts to use a multisectoral approach to control and prevent this disease.

An effective surveillance system, such as Integrated Disease Surveillance and Response, will help reduce dengue cases through timely detection of outbreaks and response strategies based on the collected information. A surveillance system with supported multisector coordination will facilitate prevention of the disease. Further, focused studies will be valuable for devising control plans.

## Appendix

Multimedia Appendix 1 Questionnaire provided to the participants of the study.

## Abbreviations

AR: attack rate
DF: dengue fever
