# Supplementary material for: Risk Factors of Dengue Fever in Urban Areas of Rawalpindi District in Pakistan During 2017: A Case Control Study
Source: JMIR Public Health Surveill. 2022 Jan 19;8(1):e27270. doi: 10.2196/27270 (PMC8811695; doi:10.2196/27270)
Supplement: Multimedia Appendix 1 [file publichealth_v8i1e27270_app1.docx]

**Annex 1:**

**Outbreak investigation of Dengue fever in urban areas of District Rawalpindi, Pakistan- September 2017**

**Questionnaire**

**Demography**

| Name |  | Occupation |  |
| --- | --- | --- | --- |
| Gender |  | Age |  |
| Contact No. |  |  |  |
| Address |  | | |

**Travel History**

| 1. | Travel to any city within last 02 weeks? | Yes | No |
| --- | --- | --- | --- |
| 2. | History of contact with confirmed Dengue case? | Yes | No |
| 3. | History of visiting hospital in last 02 weeks? | Yes | No |
| 4. | History of having fever last year during same season? | Yes | No |

**Co-Morbidities**

| Hypertension | Yes | No |
| --- | --- | --- |
| Diabetes Mellitus | Yes | No |
| Malignancy | Yes | No |
| Chronic Liver/ renal disease | Yes | No |

**Risk Factor Information**

| 1. | Presence of stagnant / dirty water wat around home/ area | Yes | No |
| --- | --- | --- | --- |
| 2. | Do you have regular water supply at your home? | Yes | No |
| 3. | If not, is water is stored in drum/ open containers? | Yes | No |
| 4. | Do you and your family members use mosquito repellents regularly? | Yes | No |
